# The Principles and Practice of Medicine; Founded on the Most Extensive Experience in Public Hospitals and Private Practice; and Developed in a Course of Lectures Delivered at University College, London

**Published:** 1840-04

**Authors:** 


### Art. IX.

1. *The Principles and Practice of Medicine; founded on the most extensive experience in public hospitals and private practice; and developed in a course of Lectures delivered at University College, London.* By John Elliotson, m.d., f.r.s., &c. *With Notes and Illustrations, by* Nathaniel Rogers, m.d.—*London,* 1838. 8vo, pp. 1088.

2. *Lectures on the Theory and Practice of Medicine, delivered in University College, London.* By John Elliotson, m.d., f.r.s., &c. *Edited by* J. C. Cooke, m.d., *and* T. C. Thompson, m.d.—*London,* 1839. 8vo, pp. 744.

3. *Clinical Lectures, delivered during the Sessions* 1834-5 *and* 1836-7. By Robert J. Graves, m.d., &c., Dublin. (*American Medical Library—Philadelphia,* 1838.) 8vo, pp. 405.

4. *Lectures on the Theory and Practice of Medicine.* By William Stokes, m.d., &c., Dublin. (*American Medical Library—Philadelphia,* 1838.) 8vo, pp. 214.

5. *Elements of the Practice of Medicine.* By Richard Bright, m.d., and Thomas Addison, m.d., Physicians to Guy's Hospital. Vol. I.—*London,* 1839. 8vo, pp. 613.

6. *Elements of the Practice of Medicine, designed as a Text-Book for the use of Students.* By William Reid, m.d.—*Edinburgh,* 1839. 8vo, pp. 573.

7. *Elements of the Theory and Practice of Medicine, designed for the use of Students and Junior Practitioners.* By George Gregory, m.d., &c. Fifth Edition; revised and considerably enlarged.—*London,* 1839. 8vo, pp. 750.

The British press has teemed, of late years, with elementary treatises and published lectures on the practice of physic. Such productions, especially the former,—being devoted to the exposition of current doctrines and precepts, rather than to the detail of individual opinion or the lengthy discussion of controverted points,—afford good materials for an estimate of the general state of the art in any country at a given time. Adopting this criterion, we think there is ground of congratulation on the late progress and actual state of medicine in the British dominions; and we would even venture to express a belief that there exist amongst us a juster appreciation of the relative importance of the several sources of the science, a minor subservience to any dominant hypothesis, and less of mechanical routine in the treatment of disease, than in any other country of Europe.

The works whose titles head the present article emanate from the three great centres of medical science in these islands: we shall, therefore, take advantage of the occasion to throw a glance over the actual state of British pathology and practice, indicating what we conceive to be its leading excellencies and defects, and the directions in which we may look for the most important additions to our knowledge; noticing, at the same time, such passages of our authors as bear most on the general argument, and commenting briefly on their merits. We have yet received only in part the lectures of Dr. Stokes, as published in the "American Medical Library;" we shall, however, make use of them as far as they go.

It is generally admitted that the rapid improvement of pathology within the last half century has been owing mainly to the increased cultivation of morbid anatomy. While it were vain to deny that the French have far exceeded us in the diligent accumulation of facts relating to this subject, we must claim for our countrymen the merit of having confined their application within juster limits, and brought them into more useful relation to medicine. In England pathologists have for the most part contented themselves with deducing from the inspection of bodies after death a knowledge of the changes of structure in which diseased actions terminate; of the precise seat of such actions and changes; and of the local complications of general affections. These are the true uses of pathological anatomy: it is the misapplication of it which produces a tendency to localize all disease, and to exclude the idea of all deranged action that does not lead directly to change of structure—a mode of reasoning which, if closely examined, will be found tantamount to regarding the animal body as a mere congeries of vessels; since it is by the disordered action of these alone that morbid alterations of structure are immediately produced. The tendency of a purely anatomical pathology is exactly the reverse of what it might at first appear. It professes to give certainty to medical doctrine, by bringing everything under the cognizance of the senses; whereas, in reality, each observed fact is forced to explain all the phenomena coincident with it, whether it have any rational relation to them or not; and thus a large and fertile tract of enquiry, where we have the inestimable advantage of subjecting facts to ocular demonstration, is converted into a hotbed for the growth of hypotheses. We regard this as one of the most unfortunate obliquities that has occurred in the course of medical enquiry; and without meaning to imply in the smallest degree that it is an universal error of French pathologists, or that our own have not frequently fallen into it, we cannot but consider the correct estimation and rational use of morbid anatomy as characteristic and happy features of British medicine. The reader will find some good remarks on the relation of morbid anatomy to medicine in Dr. Stokes's introductory lecture, which we further recommend, as an able exposition of the manner in which our art should be studied, and withal an animated and striking discourse, well adapted to win the attention and call forth the energies of the student.

The physiological study of medicine engenders a disposition to regard the diseased no less than the healthy body as a whole, the parts of which are connected by relations in many instances clearly determinate, and in others not the less real because they elude our means of research. From such a view of the nature of disease has arisen a general principle of *pathological relation*, which is, perhaps, the most important yet established in medicine, because it has reference at once to the origin, the character, and the treatment of morbid actions. It may be thus briefly stated : Certain surfaces and organs of the body are especially exposed to the extraneous causes of disease, and these surfaces and organs are precisely such as are connected with other parts, and with the whole of the living system by the most extensive sympathies and equilibria of action : a large proportion, therefore, of the true theory of disease consists in tracing the origin of morbid actions to parts remote from those in which they are developed as secondary phenomena; in distinguishing primary from consecutive disorders; and in determining the laws according to which a diseased state of one surface or organ produces the same or a different diseased state in another. The germ of this doctrine may be found in many writers previous to Abernethy, but to him we are indebted for the first attempt to expand it. His views, though distinctly based on the principle of pathological relation, were very limited in their scope, and carried to such excess with respect to a particular set of organs, that their immediate application was productive of considerable evil as well as much good.

In the hands of Broussais the principle has been more distinctly evolved, more variously illustrated, and, in many instances, happily applied to practice; but Broussais could conceive no morbid state except that of inflammation, and accordingly his doctrine is only good for as much as inflammation will explain. It may be observed also, that the data on which his reasoning is founded are insufficient, inasmuch as some of the most important inlets of disease are not taken into account. We may instance the nervous system. The noxious agents to which this system is immediately exposed are doubtless widely different from those which affect the digestive, respiratory, or cutaneous surface ; but they are not less numerous and influential. Every inordinate impression on the senses, every excessive emotion of the mind, is a more or less powerful morbific cause ; and it seems not a little singular that, while we admit the influence of acrid ingesta in causing gastric irritation, and of atmospheric vicissitudes in producing catarrh, we are apt to forget that an intense blaze of light or too loud or long-continued a sound may occasion headach; that protracted mental distress may induce disease of the heart; and that any overwhelming passion may cause instantaneous death. It would be easy to point out other directions in which noxious impressions from without are received by one organ or system, and propagated to others, or to the whole economy. But it would be out of place here to prosecute this subject further; and we will content ourselves with suggesting that, if any intelligent and well-informed enquirer would take up this doctrine of pathological relation—not with a preconceived preference for one surface, organ, or system, nor for one morbific cause or morbid action, but with an impartial reference to all that is known of the immediate and remote influence of injurious impressions on the living body—he might gain much credit, and confer great benefit on science, at an expense of labour amounting to little more than that of collecting and digesting the materials which already lie scattered over the field of medical literature.

A beneficial result of the physiological study of medicine in the present day is a growing disposition to emerge from the exclusive *solidism* which has till lately prevailed since the final explosion of the Galenic doctrines. That the blood probably possesses life, and is certainly under the immediate and extensive influence of vital agents, are considerations which must inevitably direct attention to the fluids in any system of medicine of which physiology is the basis. The superiority of a physiological over a merely anatomical pathology, as well as the importance of the fluids considered as a source of disease, is very strikingly illustrated by recent researches into the origin of morbid formations. Take for example *tubercle*. So long as we are occupied merely with looking at, handling, and incising the parts in which this morbid structure is evolved, and whose natural texture is subverted by it, we are engaged in a pursuit which bears somewhat the same relation to the study of medicine as that of simple conchology does to the natural history of the testaceous mollusca. We take up a shell, and we call it by a certain name, but we know nothing of the animal to which it belongs, and the animal is dead, or we should not now have its shell in our cabinet. In like manner, we may take up a tubercular mass, and mark minutely its texture, colour, and consistence, and we may say this is the disease called *tubercle;* but the part is destroyed, or we should not now be examining it anatomically. Now all this, though highly important as leading to better things, throws not a ray of light on the origin or cure of the scrofulous diathesis; but if we take the *blood* into account, and reflect that the essential part of the morbid formation may consist in an inorganic product deposited from it, and that the morbid mass we are examining may be composed of the natural texture of the part infiltrated with such matter, and variously incorporated with it, or obliterated by it, we then prosecute our anatomical analysis with a definite object, to ascertain whether this be actually the case; and if we find it to be so, we have made an important step in pathology, and may even entertain a hope that formidable or fatal diseases may, at some happier period of our art, be subdued or prevented by corrigents applied to the blood or to its source in the organs of assimilation. Perhaps the most interesting point in the pathology of morbid formations is their distinction into those of which the characteristic substance is organic, from those in which it is inorganic. Now, this is an essentially physiological question, and, without losing sight of the merits of Andral, Cruveilhier, and others, we think it will be conceded that the labours of Carswell have brought it out in bolder relief, and contributed more to its elucidation than those of any other enquirer.

Among the important studies which follow in the train of physiological medicine are the investigation of the laws of contagion; of epidemic and endemic influences; and of the hereditary tendency of diseases;—subjects quite out of the reach of the scalpel, and therefore neglected for the most part by mere anatomical pathologists; but which, viewed in their true light, as connecting medicine on the one hand with natural philosophy, and on the other with public hygiène, afford some of the finest pursuits to which the scientific physician can addict himself.

On the whole, we think that British medicine has always partaken largely of a physiological character, and we rejoice in the belief that it is daily becoming more firmly established on a physiological basis, which we are convinced is the only true and permanent one.

On the state of British therapeutics we may observe, that one of its peculiar excellencies resides in the conjunction of a rational empiricism with the fulfilment of scientific intentions. We employ many remedial agents simply because they are found to be beneficial, and do not object to a remedy approved by observation, because we cannot explain the mode of its action. From the absence of a like submission to experience among some of our continental brethren, several of the most efficient articles of the materia medica are hardly ever used, or altogether proscribed. Thus, colchicum is nearly excluded from French practice by objections merely theoretical; and mercury is scarcely employed except as a purgative for children;—one result of which is, that some acute inflammatory diseases are much more fatal in France than in England. It is observed by Dr. Graves, and we believe with perfect truth, that most cases of pericarditis in France die, while most of ours recover.

It must be admitted, however, that we are very apt in this country to give in to a much less laudable kind of empiricism, the result of which is an extreme degree of credulity as to the powers of particular medicines. A *reason* not being always demanded why a remedy should be serviceable, the fact that a certain number of patients get well under its use is too often esteemed a sufficient proof of its salutary effect, without due consideration of the question whether they would not have got well as fast or faster without it. We would observe also that, although the practice of British physicians is characterized in many instances by great vigour and efficiency, these advantages are seriously counterbalanced by over-activity in cases where energy is not required, and may be highly injurious; and where a due regulation of diet, regimen, and habits is all that is needful or properly admissible. In truth, we meet with daily examples in which the harmless *tisanes* of our Gallic friends, or the infinitesimal doses of the German homœopathists, might be most advantageously substituted for our own too vehement practice.

Before entering into the particular subjects of the present article, we may be expected to say something on the classification of diseases, with a view to their connected study. There can be no doubt that the high and deserved reputation of Cullen gave an undue prominence to methodical nosology, and that practical medicine has sustained little loss from the comparative neglect into which this study has fallen among the elementary writers of the present day. Of the authors before us, Dr. Elliotson thinks methodical nosology useless, and advises his pupils never to plague themselves any more about it; he nevertheless admits the advantage of a general arrangement in which morbid actions are considered as to their *nature*, and as to their *seat*. Drs. Bright and Addison express no opinion on the subject. The first volume of their work, which is all that is yet published, embraces the consideration of fevers, of inflammation in general, and of the various diseases which are either distinctly inflammatory or usually supposed to be more or less connected with inflammation in their origin. Dr. Reid follows the classification of Cullen, with such variations as are necessary to adapt it to the actual state of knowledge. Dr. Gregory, in the present edition of his work, adopts an arrangement in which constitutional or general disorders take the precedence, and are followed in succession by those of the brain and nervous system, those of the thoracic viscera, of the abdominal viscera, and of the more superficial parts of the body. Dr. Stokes reverses the order commonly pursued; and, instead of treating first of general pathological subjects, enters at once into the consideration of particular diseases, commencing with those of the digestive system. Dr. Graves's lectures, being clinical, necessarily exclude classification. If we were to advance an opinion on this subject, we should say that methodical nosology, employed as it has been to reduce all known diseases within the limits of a natural history arrangement, is worse than useless, and that all attempts at a universal classification must fail till we are perfectly acquainted with the nature, seat, and symptoms of all morbid actions: it nevertheless appears to us that the objects of pathology may be brought into very useful relations by the formation of insulated groups, formed sometimes on one principle and sometimes on another. For example, who can doubt the advantage of considering in connexion those diseases which consist in inflammation of parts whose texture is similar? Here is a nosological group formed on the ground of *general anatomy*. Again, who can doubt the advantage of regarding, in a common point of view, the diseases of the central organs of circulation and respiration? Here we have a group associated on the ground of *special physiology*. Will any one deny the utility of treating in connexion of the several exanthemata? or of grouping together the various forms of fever distinguished by periodicity of type? In each of the two latter instances we go on the simple principle of the *analogy of phenomena*, the cause or mechanism of which we do not pretend to understand: our nosology is here destitute of any scientific foundation, but the relations it establishes lead to important points of practice. The same disease may enter advantageously into the formation of several different groups, according to the point of view under which it is contemplated; and hence the impossibility of forming a general nosology on principles either philosophically true or practically useful. If these observations be just, it follows that methodical nosologists have failed from attempting too much, and have applied their science chiefly where it could be of least use—in defining the limits of systems, or forming the groundwork of elementary treatises.

In the brief remarks we are now about to make on some points of practical medicine, it is unnecessary to follow any other order than that which convenience may suggest. It is scarcely necessary to add that, amid the multiplicity of objects which present themselves, we must be content with selecting such as are most illustrative of important principles, or such as derive new interest from the observations, or are rendered imperfect by the omissions of our authors.

*Fever.* It has been very usual with writers on the practice of physic to consider the paroxysm of ague as a kind of epitome of the phenomena of fever in general, and to make it the basis of their illustration of the subject. Of the authors before us, Drs. Bright and Addison and Dr. Gregory fully admit this principle. While it is easy to recognize the affinity between ague and remittent fever, and to conceive them referrible to a common cause, variously modified in its operation and complicated in its effects, we confess it has always appeared to us inexplicable that any parallel should have been drawn between ague and continued fever. When we consider the persistent character of continued fever, and the marked periodicity of ague; the great tendency to local complication in the former, and the general absence of it in the latter; the rarity of a distinct crisis in the one disease, and the almost uniform occurrence of a particular crisis in the other; lastly, when we contemplate the difference of their morbid anatomy, and the wide diversity of their etiology and treatment, we are at a loss to discover wherein the analogy between them consists, and should almost be disposed to maintain that there is scarcely any other disease attended with pyrexia which has so little resemblance to continued fever as ague. In favour of the analogy, however, we find it familiarly asserted, that the two diseases pass into each other; that ague of a certain duration is often produced into remittent and thence into continued fever; and that the last-named disorder will subside into remittent fever and ague. We are not prepared to deny the possibility of these occurrences; all we can say is that, in a pretty extensive observation of marsh diseases we have never seen an instance of either of them. It is indeed true that ague sometimes becomes complicated with visceral inflammation, and this gives rise to severe and intractable forms of remittent fever, in which the remission can only be so called in contrast to the extreme intensity of the symptoms during the periodical exacerbations: the patient, in effect, labours under a *continuance of fever;* he has local inflammation with the attendant pyrexia, and he has moreover an ague, by the accessions of which the other evils are greatly aggravated. But such a case is widely different from what is ordinarily understood by "continued fever;" and if the local inflammation be subdued, the disease is immediately converted into simple ague. Again, we well know that the debility which attends convalescence from continued fever renders the patient extremely liable to an attack of ague in districts where the latter disease is prevalent; and this, we believe, is the real history of most cases in which continued fever is said to pass into ague. Dr. Elliotson makes a remark which may at first appear very singular, namely, that ague is very often mistaken for *typhus!* yet we are quite convinced that he is right. The truth is, ague when complicated with visceral inflammation is apt to assume an exceedingly adynamic form. There is great muscular debility, depression of spirits, and tendency to syncope; the tongue is brown, and the blood presents a loose crasis: these, however, are delusive symptoms, for a second bleeding will often be better borne than the first, and the coagulum of the blood will be firmer. Certain it is, however, that stupid practitioners sometimes call such cases typhus, and we have heard of the great prevalence of typhus in districts where, to our own knowledge, that disease was scarcely to be met with.

Our authors abstain, judiciously perhaps, from commenting at any length on the pathology of ague; indeed the subject is scarcely alluded to. In the absence of any certain light, we think physiological considerations afford a strong presumption that the morbific cause acts principally upon the spinal cord. The morbid sensations generally set in with a feeling of coldness in the course of the spine, and disordered functions of the cord are manifested by the hebetude of common sensation which, in severe accessions, almost amounts to anæsthesia, and by the deranged action of the voluntary muscles, which is more akin to distinct clonic spasm than to ordinary rigor. It is not uncommon to see the shivering of the cold stage accompanied with movements somewhat resembling those of chorea; and the same phenomena in a more intense degree have occasionally been exhibited in the various forms of convulsive ague described by authors. It may be observed, also, that those singular cases in which the ague is confined to one half of the body seem to favour the notion that the immediate seat of the disorder is in a *duplex organ*—which is probably the spinal cord, since we have no reason to believe that the brain is particularly implicated.

On the whole, we think that ague may be rationally regarded as forming a kind of link between the *pyrexiæ* and the pure *neuroses;* and that much juster and more useful analogies would spring from such a view of it than from the common one which bases the doctrine of fever on the paroxysm of ague.

A word on the treatment of ague: A variety of remedies have gained an ephemeral reputation as febrifuges, but, being weighed in the balance, have been found wanting. This, we believe, has arisen from the very different degree of curability in the sporadic and the endemic disease. In a place where ague is rare, anything may cure it which produces a momentary change in the state of the system, because it has such a strong tendency to a spontaneous cure; thus, an emetic or an anodyne given on the approach of the cold stage, a fright, or even a charm acting only on the imagination of the patient, may remove the disease: but in a marshy district, where the morbific cause is in constant and energetic operation, the lighter remedies will generally fail altogether. Remedies for ague must be tried in an ague country, or the results of observation will be very equivocal. If the practice lately advocated, of bleeding in the cold stage, had been brought to this test, we imagine there would have been very little dispute as to its claims to confidence. The authors under review seem generally unfavorable to this practice in cases of simple ague. Our own experience has convinced us that it is not generally attended with the dangerous results attributed to it by some authors, but that it is nevertheless prejudicial, by increasing the severity of the subsequent paroxysms, and protracting the duration of the disease. Whatever debilitates the body renders it more liable to ague and less able to shake it off; and in ague districts every peasant knows that if he has been bled, for whatsoever reason, he is more than usually apt to catch the ague. We beg to be understood as speaking of the disease only in its simple form: where it is complicated with local inflammation, bloodletting must be resorted to on ordinary principles; and it is a curious fact that in such cases venesection often subdues the ague along with the inflammation; more frequently, however, the ague continues, and requires the use of the bark.

There is another form of ague in which we have found bleeding very serviceable. It is popularly known by the name of *dead* or *dumb* ague. There are no distinct paroxysms, but frequent irregular shiverings; the pulse is feeble and intermittent; and the patient is constantly cold, languid, and oppressed. Here a moderate venesection, combined with measures to correct the abdominal secretions which are in a most disordered state, will often immediately change the aspect of the case; the ague assumes a regular form and type, and becomes curable by the ordinary means. Drs. Bright and Addison allude to this form of disease, but say nothing of the treatment: we direct attention to it, because it is by no means uncommon, and requires to be dealt with cautiously, for if the bark be rashly given it may occasion visceral inflammation; indeed we have seen this form of ague converted into a most anomalous and intractable remittent, by the premature use of febrifuge medicines.

Of the only two febrifuge remedies of established reputation, bark and arsenic, our authors generally give the preference to the former; nor can we at all agree with Drs. Bright and Addison, when they represent arsenic as "a remedy rivalling if not actually surpassing the cinchona in power." We never saw the bark fail (in the shape of sulphate of quinine) in a single instance in which it appeared to us to be properly applicable and judiciously used; but arsenic has very often disappointed us entirely, or has not succeeded till pushed to a dangerous dose; and we are bound to add that we have too frequently known the exemption from ague purchased at the price of a very serious irritation of the gastro-enteric membrane. Dr. Elliotson says he has failed with arsenic, but never with bark. The cases in which we have found arsenic most useful are those in which there is a great tendency to relapse. Quinine cures the ague when present, and will generally prevent a paroxysm when threatened, but it seems to have little power of obviating the morbid disposition: if, however, the quinine be given to arrest the disease, and the arsenic be then exhibited in moderate doses, and continued for some time, the morbid tendency will generally disappear.

There has been some discrepancy of opinion among authors as to the propriety of administering bark in ague when inflammation is present. The common precept is not to use this medicine when there are any marked symptoms of local inflammation or congestion, and we believe it to be perfectly just. Several writers of authority, however, have deviated from this rule, and Dr. Elliotson appears to do so in the following passage, which, however, is not remarkable for perspicuity:

"It was formerly thought very wrong to stop an ague until the patient had gone through a certain preparation. I have surprised many persons who, when in other countries, used to see some preparation employed before the remedies for ague were given, by stopping the ague immediately. I never saw injury arise from it; though, if there be any local affection of the head, chest, or abdomen, you must be cautious, and attend to that at the same time. Should there be any congestion of the head, lungs, or abdomen, it may be necessary to bleed, purge, and use all the other remedies for this state; for if you do not, it is possible that the mere stopping the ague at once may be useless. When you have done everything indicated by the local affection, there will be no danger in stopping ague. I never lost a patient by employing sulphate of quinine when local inflammation was present. Arsenic may be very improper when the inflammation which is present affects the stomach; and in the case of gastritis, probably neither arsenic nor quinine can be borne; and you do not remedy the morbid condition till you adopt local or general bleeding. I have seen local disease removed the more easily after stopping ague; for every paroxysm, of course, disturbs the circulation, renders it more irregular, and is likely to throw a greater load of blood upon those organs which are in a state of congestion. At any rate, ague will make bad worse." (*Ed. Cooke and Thomson,* p. 173.)

Dr. Elliotson, if we understand him aright, believes that the ague generally *can* be stopped, notwithstanding the presence of the local affection. Here we differ from him. We have seen this happen in a few cases; but in a great majority we have found that the ague has been rendered more violent by the aggravation of the local disease: in many instances, also, the type of the ague has become confused, and the case has passed into one of severe and intractable remittent fever. The treatment of ague accompanied with inflammation or congestion requires much caution, and the life of the patient sometimes depends on the vigilance of the practitioner. If we can get the inflammation under, so as to secure a single distinct intermission, or a near approach to it, we can generally control the disease. A single dose of the sulphate of quinine should be given, and its effects carefully noted: if it produce any considerable return of local pain or febrile excitement, we must not repeat it, but wait for a better opportunity; if no bad symptom occur, the medicine should be pushed boldly; and if there be no accession of ague at the next period it may be continued with confidence of success.

It occasionally happens that ague is complicated with some slight inflammatory affection, which shows itself *only during the paroxysms.* Here also it is better to get rid of the local disease first, if we can; but if it prove obstinate, the quinine may be given in the intermissions, and the ague and inflammation will most likely cease together.

We are happy to find that none of the writers before us, in treating of *continued fever,* embrace any of those hypotheses which regard local inflammation as its essence; although we are by no means disposed to deny the frequent occurrence of this state as an incidental complication. With respect to the local derangements of vascular action occurring in the course of fever, Dr. Reid expresses a strong doubt whether they be identical in character with common inflammation, and is inclined to believe that they may more properly be regarded as peculiar results of disordered innervation. He observes, that if the nervous influence be weakened or deranged, a change may be effected in the relation of the fluids to the textures through which they circulate, whereby the ordinary stimulus of the former produces morbid effects, just as in a similar state of the nervous system the ordinary stimulus of light or sound occasions pain and disturbance in the organs of sight and hearing. He adds, that too little attention has hitherto been given to the enquiry, how far the universal derangement of one class of functions modifies subordinately the disturbed conditions of others. We think these remarks worthy of consideration. We have long been of opinion that disordered innervation is much concerned in the local congestions alluded to, and have on former occasions adverted to this topic. Many arguments might be adduced to prove that such congestions, in many cases at least, differ materially from ordinary inflammation, but the one on which we would principally insist is the length of time which they may endure without causing change of structure. Thus a fever patient shall labour for many weeks under a state of brain supposed to be inflammatory, and yet he shall be restored to perfect health without a vestige of any organic lesion within the cranium. Another shall be affected for the same length of time with what is conceived to be gastro-enteritis, yet this patient also shall recover entirely. In each of these cases, moreover, the transition from a state of dangerous disease to that of complete convalescence may be exceedingly sudden. Now we can hardly believe that real inflammation of a pretty active character could often exist so long without committing fatal ravages, or at least leaving behind it vestiges which would long be perceptible. We dwell for a moment on this point, because, if there be any truth in the opinion advanced, an important practical corollary follows, namely, that the practitioner must not expect to get rid entirely of such complications of fever, but must content himself with keeping them under, trusting for their final removal to the cessation of the general disorder, of which they are merely the effects. The most disastrous results often arise from an obstinate determination to subdue such local affections by repeated bloodletting: the topical disorder is not subdued, and, what is worse, the vital powers of the patient are irretrievably prostrated.

We by no means contend that fever is not frequently complicated with actual inflammation, or that the early and prudent use of the lancet in such cases is not advisable; here, however, the fever is usually of a distinctly inflammatory type. The *insidious* inflammation, as it is called, which accompanies low fever is, we believe, distinct from common inflammation occurring in the same textures, and is not to be subdued by the same means, although the occasional application of a few leeches will often suffice to keep it below the point of disorganization.

Drs. Bright and Addison treat, under distinct sections, of fever with predominant cerebral, abdominal, and thoracic affection, and of the modifications of treatment in each. This is judicious, and will tend to give greater distinctness to the ideas of the student, who will find no difficulty in combining them when more complex cases present themselves. There is one point to which we would here advert, which has probably attracted the attention of most practitioners, but which is not usually mentioned in elementary books: we mean the occasional difficulty of ascertaining whether the local affection complicating fever be seated in the abdomen or in the head. We shall presently, however, have occasion to notice this subject when speaking of abdominal diseases, and have now only to remark that the observations there made are no less applicable to fever than to other acute disorders.

In the treatment of continued fever there is a question of considerable moment, on which the best authorities differ, namely, how far it is possible to *cut it short.* Dr. Elliotson speaks of arresting fever *in limine* as a frequent occurrence; Drs. Bright and Addison as an occasional one; Dr. Gregory regards it as so rare that the hope of it cannot be made a foundation of rational treatment; and Dr. Reid, in expressing a similar opinion, suggests that as it is by no means easy, except in very well-marked cases, to pronounce on the probable duration of fever, supposed cases of fever cut short may have been merely ephemera. There may be some truth in Dr. Reid's supposition; but we think a much more frequent source of error consists in mistaking symptomatic for idiopathic fever, and imagining that we have cut short a fever when we have merely subdued an inflammation. This is especially true with respect to inflammation of the small intestine, the local indications of which are often obscure, and the symptomatic fever typhoid. Dr. Reid doubts whether it be possible to abridge the course of fever arising from specific contagion, and adduces the analogy of variola and scarlatina, which, he says, can never have their duration altered: we have, however, in one instance seen scarlatina distinctly cut short by venesection. Without denying the possibility of cutting short fever, we regard the occurrence as so rare that it may, in a practical point of view, be put nearly out of the question; neither can we help suspecting that the means employed for this purpose have frequently the opposite effect of protracting the disease. The measures most generally adopted are bleeding and emetics. Now we are convinced that the former, applied with the object of subduing fever—apart from inflammation, congestion, plethora, or other state affording a special indication—has merely the effect of somewhat debilitating the patient, and thereby somewhat retarding those processes by which nature effects the restoration of health. With respect to emetics, we have very small faith in their power of cutting short fever, while we feel well assured that they increase irritation of the gastro-enteric membrane where it is present, and sometimes determine it where it did not previously exist. It is admitted on all hands that when there is any tenderness of the abdomen emetics should not be given; but we would ask, is the absence of tenderness of the abdomen a certain criterion that the mucous membrane of the intestines is sound? We think not. In ileitis, the most frequent abdominal affection complicating fever, the tenderness on pressure is hardly ever great, and sometimes so obscure that it cannot be distinguished from the slight uneasiness caused by pressure when the intestine is merely distended with gas. We would ask also if it be prudent, when the circulation is universally deranged, and every secreting surface disordered, to adopt a measure which necessarily occasions a sudden and violent flow of blood to the intestines? From these considerations we agree with Dr. Gregory—in opposition we allow to the opinion of high practical authorities—that a foul state of the stomach affords the only proper indication for the use of emetics in fever. When this organ is loaded with excessive or acrid ingesta, as indicated by oppression, nausea, and bitterness in the mouth, an emetic may be given with advantage; and since ipecacuan evacuates the stomach as effectually, and irritates the digestive mucous membrane much less than emetic tartar, it is we think greatly to be preferred. We speak here only of the common forms of fever, for in those rarer varieties, which have obtained the name of *congestive*, and which have many points of resemblance to malignant cholera, strong emetics are among the most efficient means of rousing the circulation: nor do the objections above stated apply in such cases; for our object is at all hazards to bring out a disease that can be dealt with, and to prevent the system from collapsing into death without an effort.

We have said nothing of the cold affusion as a means of arresting fever. It is a remedy of which few practitioners of the present day can speak from large experience; but the fact that it has fallen so entirely into disuse warrants a strong presumption that it has not realized the expectations awakened at its first introduction.

Another point of great importance in the treatment of fever is the management of that state of delirious and sleepless excitement which frequently supervenes in the advanced stage, usually from the eighth to the twelfth day. Drs. Bright and Addison tell us that when active delirium comes on at this period, so that the patient becomes violent, talks incessantly, and attempts to get out of bed, the tongue being dry and brown, the skin parched, and the pulse frequent and sharp, some form of depletion is indicated, while cordials induce a state of stupor and low delirium soon terminating in death. They add that if instead of the excited state above described, there is merely confusion or stupor, depletion is still necessary. They do not recommend full bleeding, but state that the abstraction of six, eight, or ten ounces of blood is frequently followed by rallying of the vital powers, subsidence of delirium and a softer state of the pulse. We believe the applicability of these remarks will vary according to the character of the epidemy, for the cerebral affection under consideration is common to the worst forms of typhus and the synochus which is now the most prevalent form of fever in England. Taking fevers as they go, however, our own observation is not so favorable to blood-letting under the conditions alluded to as that of Drs. Bright and Addison: we have seldom found bleeding when very moderately used produce any marked effect one way or another; but when employed less cautiously we have often seen it annihilate at once all hope of recovery. When speaking of the maculated fever of Dublin, Dr. Graves expresses his conviction of the entire inefficacy of depletion in the severe cerebral affection of the advanced stage, and recommends, as a most valuable remedy, the combination of emetic tartar with opium. He gives some very striking cases in which its effects seemed almost magical. This practice Dr. Graves claims as exclusively his own, and assuredly if it prove as successful in the hands of others, he will be entitled to much gratitude for its introduction. The subject of continued fever is well handled by the authors under review. Drs. Bright and Addison have given a neat and flowing outline of it. Dr. Reid has entered into it very much in detail, and viewed it in a great variety of bearings: it is just to add that he has given many indications of a philosophical mode of thinking and of close personal observation. Some of the clinical remarks of Dr. Graves are extremely valuable; and Dr. Stokes's observations on abdominal inflammation have also an interesting relation to the subject.

For an excellent and comprehensive account of the *exanthematous fevers*, we have great pleasure in referring the reader to the work of Dr. Gregory from the thirteenth to the twentieth chapter inclusive.

*Diseases of the Abdomen.* No department of medicine has derived greater light from morbid anatomy than that which relates to abdominal diseases and the various remote affections dependent upon them. The discovery of the mucous form of enteritis, for which we are indebted to Pemberton, and which has unveiled the immediate cause of many serious visceral diseases and the chief source of danger in some of the worst forms of fever, would alone constitute an era in the history of medical theory and practice.

In our comments on abdominal disease we shall chiefly follow Dr. Stokes, whose lectures on this subject are truly admirable, and evince so judicious an union of the views of Broussais with those of our own countrymen, so ample an experience, and so consummate a tact in discerning and treating disease, that we cannot recommend them too strongly to the student. In treating of *gastritis*, Dr. Stokes dissents from the opinion of Broussais that idiopathic inflammation of the stomach never occurs but in connexion with a similar state of the small intestine; he admits, however, the extreme rarity of pure gastritis, and attempts to account for it on the physiological principle that the stomach is an organ, the healthy functions of which require that it should be frequently in a state of great vascular excitement, and hence that it is adapted by nature to sustain with impunity an influx of blood, which would cause inflammation in another organ—otherwise every act of digestion would be followed by gastritis: the idea is ingenious, and as far as we know original. Dr. Gregory seems scarcely to admit the existence of gastritis, except as a peculiar affection of infants, and as the result of habitual drunkenness or poisoning. Drs. Bright and Addison consider it as a rare disease; Drs. Elliotson and Reid say nothing as to the frequency of its occurrence; the former, however, remarks that the whole of the inner surface of the stomach is seldom found inflamed, but only some particular part, and this corresponds with our own observation. We should say that partial gastritis is not at all uncommon, while universal gastritis is very rare, though not so much so as some authors would lead us to believe. We may add that we have sometimes found on dissection a limited portion of the gastric membrane inflamed in cases where there had been no symptoms during life to lead to the suspicion of such disease.

Dr. Stokes points out the error into which Abernethy and Broussais have led their followers by representing the state of the tongue as an unerring guide to that of the digestive tube. In opposition to this he cites the extensive observations of Andral and Louis on the state of the tongue in the gastritis of continued fever, from which they infer that there is no constant relation between the state of the tongue and that of the stomach. Dr. Stokes's experience has led him to a similar conclusion with respect to the idiopathic affections of the intestinal canal; and he considers the appearance of the tongue as of much more importance as an index to the condition of the general system than to that of any particular organ. On the whole we believe this opinion to be just. We are at all events quite convinced that serious disease of any part of the digestive apparatus may coexist with a natural state of the tongue. Of this truth, with reference at least to the stomach, we recently met with a striking example in a female patient, who had suffered for several years under an increasing affection of this organ, and who when she first applied to us had constant tenderness of the epigastrium, and acute pain at the cardiac orifice in the act of swallowing, with entire loss of appetite, debility, anxiety, and some emaciation—symptoms indicative of no very sound condition of the stomach: nevertheless this patient's tongue might have been selected as an example of a perfectly healthy one.

Dr. Stokes illustrates very ably the sympathetic affections which are liable to supervene on inflammation of the intestinal tube, especially in its upper portion, and directs attention to the important law that when such inflammation has subsisted for some time, and attained a certain degree of intensity, the original local symptoms may subside altogether, and the gastritis or enteritis be represented by the symptoms of disease in the organ secondarily affected, as the brain or lung, and yet the primary inflammation shall exist all the while in full force. He cites two illustrative cases. One is from Andral: the symptoms of gastritis were suspended on the supervention of tetanus which was speedily fatal; and on dissection no morbid appearances were found in the brain or spinal cord, but the inner surface of the stomach was intensely inflamed. The other case occurred in the Meath Hospital: the patient on admission was labouring under violent maniacal excitement, with bloodshot eyes, a ferocious aspect, dry, fissured tongue, quick, weak pulse, and constipated bowels; there was no tenderness of the epigastrium, no vomiting or other symptom of gastritis, though on the third day the belly was slightly tender and tympanitic; the cerebral symptoms increased, and death took place on the eighth day: on dissection extensive inflammation of the digestive tube was discovered, but none in the brain or its membranes. We well remember a case which we saw incidentally some years ago in one of the London Hospitals. The patient, a young woman, was lying in a state of profound coma, with fever and a rapid pulse. Being asked our opinion as to the nature of the case, and learning that there had been diarrhœa at the commencement, and that the cerebral symptoms had come on suddenly, and detecting obscure indications of pain in the abdomen from the uneasy movement of the patient when it was strongly pressed, we declared our belief that the intestines were the primary seat of disease. The eminent practitioner under whose care the patient was differed from us, adding that we should soon have an opportunity of ascertaining, as he had no doubt she would die. A few days after he told us that on dissection the brain had been found healthy, and the mucous membrane of the intestines highly inflamed.

If abdominal disease be sometimes masked by cerebral symptoms, it is no less true that affections of the brain may be accompanied at their commencement with extreme irritability of the stomach and bowels, as indicated by copious vomiting and purging. Of this every practitioner must have met with examples in the early stage of acute hydrocephalus. Dr. Graves notices a similar occurrence in scarlatina and typhus, and makes some excellent remarks on the diagnosis of such cases. The cerebral diarrhœa and vomiting generally set in at a very early period of the disease, as the first or second day, and are seldom attended with the red and furred tongue, bitterness of the mouth, burning thirst, and epigastric tenderness, which denote gastro-enteric inflammation; they are not relieved by leeching the abdomen; and they are particularly characterized by the discharge of bile which is much greater than ever occurs in gastro-enteritis. The last-mentioned symptom is very remarkable and, as Dr. Graves truly observes, is not confined to febrile affections, but occurs generally in vomiting from deranged cerebral circulation; thus, in sickness produced by swinging or sailing, immense quantities of bile are thrown up. From these considerations it is evident that in all fevers which set in with vomiting and purging which there are no abdominal symptoms to account for, the practitioner ought to keep a very watchful eye on the state of the brain.

With respect to the treatment of gastritis, Dr. Reid is the only one of our authors who recommends large venesection as a general practice. He admits, however, in common with Drs. Elliotson, Bright and Addison, and Stokes, the great efficacy of leeches. Dr. Stokes advocates strongly the internal use of ice, and advises a portion the size of a walnut to be kept in the mouth till its angles are melted down, and then swallowed whole, and this to be repeated as often as the patient desires it. Drs. Bright and Addison, and Dr. Elliotson, also recommend the internal use of ice. Dr. Stokes observes that the fact of gastritis being occasionally produced by swallowing cold water or ice when the body is heated by exercise is no argument against the use of these substances when the inflammation actually exists. Dr. Elliotson remarks, too, after Currie, that the production of inflammation by the application of cold to the stomach after exercise does not arise from the person's being *hot*, but from his being *exhausted*—in which we believe there is much truth. The internal use of cold water and ice in gastric inflammation is not yet generally familiar to British practitioners, which is much to be regretted, for it is one of the most powerful means of cure, and conjoined with abstinence and rest is often sufficient, in slight cases, without any other remedy.

Dr. Stokes makes some interesting remarks on *hematemesis* and *delirium tremens* in connexion with acute gastritis. Hematemesis, he observes, may arise from various causes; it may be vicarious; it may be accidental, as from the rupture of a blood-vessel; or it may result from mechanical obstruction of the circulation: but there is a species of gastritis of which copious hemorrhage is a prominent symptom, or, in other words, an hematemesis of which gastritis is the immediate cause. In such a case, besides the vomiting of blood, there is tenderness of the epigastrium, fever, heat of skin, thirst, and longing for cold drinks. Here the use of astringents so commonly resorted to in hematemesis is highly injurious and may be fatal: the inflammation must first be allayed by leeches and cold drinks, and if the hemorrhage still continue, astringents may then be used to check it. We would particularly direct the attention of the reader to this point, because the case here described is by no means a very rare one; yet, as far as we know, Dr. Stokes is the only author by whom it is distinctly stated. Drs. Bright and Addison remark that a profuse discharge of dark blood from the stomach sometimes occurs in chronic gastritis, especially that variety of it arising from intemperance; but this is a different case, the hemorrhage here proceeding from a relaxed and congested, or, in a few instances, an eroded state of the vessels.

With respect to *delirium tremens*, Dr. Stokes lays great stress on the two opposite conditions under which it is known to occur—after a debauch, or on the sudden suspension of the habitual use of alcoholic liquors. In the first case he believes the pathological state to consist in gastritis, accompanied with high excitement of the brain and nervous system, in consequence of the absorption of alcohol, or from sympathy with the stomach, and tending strongly towards inflammation of the brain. He considers this state as illustrative of the law just now adverted to, that inflammation of the stomach may be entirely masked under the sympathetic irritation of other organs; for the abdomen may not be tender, nor the tongue red, and all the symptoms may point to the brain as the seat of disease, and yet intense gastric inflammation may be going on all the time. The treatment is that of gastritis, and our author states that it has been used with extraordinary success in the Meath Hospital, where the most aggravated symptoms of delirium tremens have been subdued by leeches to the epigastrium and iced water. On the other hand it has been found in the same institution that in patients who died under the stimulant treatment, inflammation existed either in the stomach, or in the substance or membranes of the brain. In the second case the functions of the brain are disturbed by the abstraction of an accustomed stimulus, and the indication is to restore that stimulus: it is here, according to Dr. Stokes, that the ordinary practice of giving porter, wine, brandy, opium, &c., is appropriate and successful. From the foregoing considerations Dr. Stokes lays it down, as a general rule, that when delirium tremens arises from the want of the habitual stimulus, it is to be treated with stimulants and opiates; but that when it supervenes on an excess, the proper treatment is that of gastric inflammation. We are content to recommend these views to the careful attention of the reader without venturing on an opinion as to their general validity, for we have not had sufficient opportunities of dissection in such cases to enable us to form a decided judgment. We may remark, in passing, that admitting the pathology here laid down, we should still think considerable caution necessary in the use of depletion; for gastritis occurring in a drunkard, independently of delirium tremens, will not bear very active antiphlogistic treatment, and we are quite sure that we have seen cases of delirium tremens following a debauch destroyed by the lancet as well as others arising under the opposite condition.

Dr. Stokes's remarks on *chronic gastritis* are deserving of diligent perusal. On the whole we think he is perhaps disposed to refer too large a proportion of the cases ordinarily termed *dyspeptic* to an inflammatory state of the stomach; yet there can be no doubt that the cases to which this view justly applies are very numerous, and that incalculable mischief is continually done by the senseless use of purgatives and bitters, where a careful regulation of the diet, abstinence from all medicine by the mouth, leeches to the epigastrium, and the use of enemata to keep the bowels regular, would soon restore the patient to health. Dr. Stokes has also great reliance on counter-irritation effected by small blisters; a mild form of the tartar-emetic ointment; or the external application of croton oil, in the quantity of five or six drops, rubbed on the epigastrium, with a piece of lint or bladder interposed between the finger and the patient's skin. By the last-mentioned process an eruption of small papulæ is soon produced, which may be increased at will. Dr. Stokes observes that the action of the croton oil, as a counter-irritant, depends on its not being absorbed, for if it be absorbed, it will purge instead of irritating the skin.

In a few remarks on *cancer of the stomach*, Dr. Stokes expresses great doubt as to its true nature, and the validity of any distinction between this and common ulceration in various other parts. We entertain no doubt that the orifices of the stomach are sometimes the seat of genuine cancer—by which we understand a morbid induration, consisting of a fibrous structure, the interstices of which contain a softer matter, the whole tending to malignant and incurable ulceration. We nevertheless believe that many cases of alleged cancer of the stomach, as well as of the rectum, have no title to that denomination, and are in effect mere instances of induration and ulceration of the parietes of a muscular canal from chronic inflammation commencing in the mucous coat, precisely analogous to the state of the urethra in cases of inveterate stricture. We cannot agree with Dr. Stokes that "in the present state of medicine we are not possessed of any data which would enable us to come to a final determination of this question." We apprehend that the case is one in which diligence of observation is the thing chiefly needed, and that any pathologist who had sufficient opportunities might confer a benefit on the science, by ascertaining in how many out of a large number of cases called cancer of the stomach, the true anatomical characters of carcinoma could be made out. We admit that it is not ascertained whether a peculiar matter be deposited in cancer as in tubercle and melanosis; but still we think the morbid formation, as a whole, has sufficiently distinctive characters. However this may be, we fully agree with Dr. Stokes, that the treatment best adapted to relieve suffering and prolong life, in the cases called cancer of the stomach, is that applicable to chronic gastritis. The case of the Emperor Napoleon, as described by Dr. Stokes, affords a painfully vivid delineation of the loose diagnosis and empirical practice too common in such diseases.

"He died with extensive ulceration of the stomach, which, of course, was called '*cancerous*,' and there were also distinct traces of disease in the liver, the mucous coat of the intestines, and the lungs. His disease was believed by himself to have originated in the stomach, and to this opinion he adhered, notwithstanding the results of some solemn consultations, at one of which his affection was declared to be an '*obstruction of the liver*' with a '*scorbutic dyscrasy*.' At another it was pronounced to be a '*chronic hepatitis*,' and a course of mercury recommended! When we reflect on this, and read in the account by Gaubert, (which you will see in the *Examen des Doctrines Médicales*,) the regimen which was used, you will not wonder at the words of this great man, when he was pressed to take more drugs, to swallow the universal nostrum mercury, to which he had the greatest aversion. 'Your disgusting preparations are good for nothing. Medicine is a collection of blind prescriptions, which destroy the poor, sometimes succeed with the rich, but whose results are more injurious than useful to humanity.' But he got mercury notwithstanding, mercury for his 'digestive organs,' to 'excite the liver,' to 'remove its obstruction,' and mercury to create bile, and purgatives to remove it; and tonics and antacids, and stimulants; and he died in torture, and his body was opened, and the stomach was found '*cancerous*.'" (p. 59.)

But we must proceed to other forms of abdominal disease. The diagnosis of *ileitis* may be cited as an example of the value of negative evidence. Independently of the general indications of abdominal inflammation, the peculiar symptoms of acute *gastritis* are vomiting and the desire for cold drinks, while inflammation of the *large intestine* is characterized by tormina, tenesmus, and copious vitiated secretions. In *ileitis*, on the other hand, though there be thirst, there is no particular preference for cold drinks; indeed the patient usually prefers them warm: diarrhœa may be present or not, but the tenesmus and griping of colonitis are absent; in the latter disease also, the excretions are always highly vitiated, while in inflammation of the small intestine they are extremely various, and sometimes perfectly healthy. Gastritis and colonitis are attended with acute pain, though of very different character, while in ileitis the patient seldom complains of pain at all, though there is a slight tenderness on pressure over the situation of the inflamed intestine, especially between the umbilicus and crista of the ilium on the right side. In ileitis there is generally a tympanitic state of the abdomen. Ileitis is always accompanied by fever, and this is frequently of the typhoid type; indeed, as before observed, many cases called continued fever are nothing more nor less than simple inflammation of the small intestine. This point is properly insisted on by Dr. Stokes, whose remarks on ileitis are highly practical and instructive.

We are somewhat surprised to find Dr. Stokes among the number of those pathologists who consider *tabes mesenterica* as essentially connected with enteritis. We think also that his notion as to the prevalent pathology of the former disease is incorrect. He says, "the common idea formerly entertained with respect to this affection, and I believe still to a great extent is, that the disease first commences in the mucous glands, and from these extends to the lymphatic ganglia of the mesentery, which, in their turn, become enlarged, thickened, and less pervious, so that a sufficient share of nutriment cannot be absorbed, the consequence of which is that the patient dies of atrophy and exhaustion." We conceive that ever since tubercular matter has been generally recognized as a distinct morbid product, the current pathology of the disease in question has been that the mesenteric glands become tuberculated, under circumstances parallel to those in which other glands pass into the same state; and that, as in other cases, the deposition of the morbid matter may be preceded by inflammation or not, though the development of the diseased structure must necessarily, at some period of its progress, be accompanied by it. Let us examine the grounds on which Dr. Stokes embraces Broussais's pathology of tabes mesenterica.

"What is the actual state of the science with respect to this disease? It is found that the glands are certainly changed in their structure, and that they are manifestly enlarged; but this is only a link in the chain of phenomena, for it has been proved that in *the majority of cases the disease is ushered in by enteritis, and that the swelling of the glands is the result of disease, propagated along the course of the lymphatics, from the mucous surface of the intestines to the mesenteric ganglia.* This preparation, which I shall send round, will give you an idea of the actual state of the disease. Here is one of the glands which has been cut through; it exhibits the cheesy texture commonly observed in this disease, but you can perceive there are a number of lines running towards each of the glands; these are the engorged lymphatics, which you see correspond with ulcers on the mucous surface of the small intestine. That this is the true pathology of the disease will appear from the following circumstances:—First, it has been proved that the glands of the mesentery commonly become inflamed, enlarge, and suppurate in cases of inflammation of the mucous membrane of the intestinal canal in the adult. A patient gets enteric inflammation and dies; on dissection we find distinct marks of disease in the intestines, and, in addition to this, the glands are evidently diseased. Here is one fact. In the next place it has been proved that, in a great many cases of tabes mesenterica, if you retrace the history of the disease, if you go back to its first and earliest phenomena, you will find that it began with the symptoms of what has been termed remittent fever, or that the patient had enteritis or diarrhœa, which afterwards became chronic, and that then the symptoms of tabes mesenterica began to appear. In the third place you will find that, in a vast number of cases where a fatal termination has occurred, if you pursue your dissection, and slit up the whole of the ileum, you will discover numerous old ulcerations of the mucous membrane, and find that the lymphatics which correspond with these ulcerations are in a state of manifest disease. Lastly, it has been observed that the best treatment for tabes mesenterica is that which is calculated to remove enteric inflammation, and that the old treatment, founded on the principle of removing obstruction, by the use of alkalies, absorbents, and solvents, is erroneous and false in the majority of cases. So that we have proof of the origin of this disease in intestinal inflammation, drawn from the occurrence of analogous affections in the adult, from the phenomena of the disease in its early stage, from morbid anatomy, and from treatment. I think there can be no doubt that, in most instances, it commences by intestinal inflammation. Of course a predisposition to disease of the glandular system will favour the occurrence. But is there no case in which the disease has commenced in the glands, and where the mucous membrane of the digestive tube is secondarily engaged? My answer to this question is, in a few cases we cannot prove that the disease commenced in the mucous membrane, and there is no reason why the glands of the mesentery should not be liable to primary tuberculous or scrofulous deposition, as well as those of any other part of the body; but in a vast number of instances, the enlargement of the mesenteric glands is secondary, and resembles the inflammation of the inguinal glands which results from chancre on the penis."

The opinion that disease of the mesenteric glands is an ordinary accompaniment of mucous enteritis in adults is neither sanctioned by the accounts of the best pathologists, nor, we think, by common observation. These glands, doubtless, occasionally become inflamed and suppurate in enteritis, and especially where it is a complication of continued fever, but we do not hesitate to affirm that the occurrence is comparatively rare. Moreover, admitting the universality of such lesion in enteritis, we cannot perceive the analogy between suppuration of a gland from acute inflammation, and the changes which take place from the deposition of tubercular matter in its texture; and that tubercular matter *is* deposited in the mesenteric glands in the majority of cases of tabes mesenterica, we hold to be as certain as any truth in pathology. It is spoken of as a simple matter of fact by Andral, Carswell, Sir James Clark, and many others; and if the pathologists we have named do not know tubercular matter when they see it, there is an end of the study of morbid anatomy. The mesenteric glands in the disease in question present exactly the same appearance, and their texture is infiltrated with the same matter as tuberculated bronchial glands; and we will answer for it that if two such diseased glands, of equal size, the one bronchial and the other mesenteric, were insulated, and presented to the best morbid anatomist in Europe, he could not tell one from the other. We do not deny the frequent coincidence of inflammation of the intestinal membrane with tuberculated mesenteric glands; but the observation of Andral has established that a similar state of the bronchial membrane is equally frequent when the bronchial glands are tuberculated, and in the latter case inflamed absorbents are out of the question as a cause of the glandular affection, because, of the lymphatics which enter the bronchial glands, very few come from the mucous membrane of the bronchi, since they are almost all derived from the peripheral surface of the lungs, or creep along the outside of the bronchial tubes from their extremities. It is very probable, however, nay there is little doubt, that in both instances the occurrence of inflammation in the mucous membrane may determine the specific morbid action in the contiguous glands already predisposed to it, just as bronchial inflammation may stimulate into morbid activity the rudimentary tubercles which have long lain dormant in the substance of the lung.

We must further observe that disease of the mesenteric glands does not appear to be so frequent a sequel of the infantile remittent fever, or other forms of enteric inflammation, as Dr. Stokes's doctrine would lead us to suppose. We admit that it often occurs; but not, we think, much oftener than other strumous affections, as enlarged lymphatic glands, chronic abscesses, and diseased joints, which we agree with Drs. Bright and Addison in regarding as manifestations of a general scrofulous diathesis, occasioned by the continuance of febrile irritation, and determined as to their particular form and locality by individual predisposition. But after all, the observations of Broussais, and Henning, and Macintosh, and Stokes are not to be unceremoniously set aside, however apparently opposed to those of great pathologists; and we suspect the truth will turn out to be that, independently of the common tubercular form of tabes mesenterica, there is an inflammatory enlargement of the mesenteric glands in children consecutive on inflammation of the enteric mucous membrane, analogous to that first described by Petit and Serres, as an accompaniment of a fever occurring in Paris in the years 1811-13, and since repeatedly observed. Pemberton has described an enlargement of the mesenteric glands distinct from tubercle, arising in consequence of chronic peritonitis. "It frequently happens," says he, "that the mesenteric glands are enlarged, but I never saw suppuration in them or cheesy matter; so that I consider it a very different disease from a scrofulous affection of these glands, as it seems to be an enlargement of them, merely from irritation of the inflamed membrane with which they are surrounded."* Other writers, however, have described a tuberculated state of the mesenteric glands as frequent in chronic peritonitis.

It is evidently of much practical importance to ascertain whether there be indeed two distinct forms of disease in the mesenteric glands common among children: for assuredly the treatment of scrofula is not that adapted to enteritis, nor would strumous children derive much benefit from being treated, during a protracted state of atrophy, for an enteritis which had no existence, or was at most incidental and subordinate.

Our space will not allow us to follow Dr. Stokes through his delineation of the diseases of the large intestine. We should not, however, be justified in omitting to notice his observations on a particular form of diarrhœa connected with partial ulceration.

"It not unfrequently happens that a person, labouring under chronic diarrhœa, comes to consult a medical practitioner, and tells him that he has been suffering from this complaint for months, that he has eight or nine discharges by stool in the day, and that he has been under the care of five or six doctors in succession, without any benefit. Well, you are determined to have your trial, too, and you commence operations by putting him on full doses of acetate of lead. After a week or a fortnight, he comes back and tells you he is not a

* On Diseases of the Abdominal Viscera. Second Edition, p. 13.

bit better. You then try turpentine or balsam of capaiva—no use. Nitrate of silver—the same result. The man gets tired of you in turn, and perhaps goes to a surgeon to ask his advice. The surgeon examines the rectum carefully, and finds, at a short distance from the anus, an ulcer, which he immediately touches with a strong solution of nitrate of silver. The ulcer begins to heal, the irritation of the gut ceases, and the diarrhœa goes off. The surgeon is extolled to the skies, and the doctors disgraced for ever in the opinion of the patient. Now this is not an uncommon case. I have seen several instances of it, and I must tell you I was once mistaken in this way myself. These ulcers are situated close to the verge of the anus; they occur chiefly in persons of broken-down constitution, and those who have taken a great deal of mercury. They produce irritation in the colon, tenesmus, griping, frequent discharges by stool, and most commonly during the straining, a little blood is passed. During the course of last summer, I treated a soldier for this affection, who had been discharged from the East India Company's service (as was stated in his discharge) for incurable dysentery. I examined the rectum, and finding some ulcers close to the anus, had them touched with the nitrate of silver. Under this treatment a rapid amendment took place, and in the space of three weeks the man was discharged, quite cured. Now, are you to make this examination in every case? I believe you will act rightly in doing so in every case of chronic diarrhœa in the male, but the examination is absolutely necessary in all cases under the following circumstances: first, when the diarrhœa has been of long standing; secondly, when it has resisted a great variety of treatment; thirdly, when it has been combined with tenesmus and a desire for sitting on the night-chair after a stool has been passed, showing irritability of the lower part of the great intestine; and, lastly, when the patient's health does not appear to be so much affected as it naturally should be, where there was long-continued disease of a large portion of the great intestine. A patient will come to consult you, who will inform you that he has had eight or ten alvine evacuations every day for the last six months, and yet he eats heartily and looks quite well. Under these circumstances, the cause of the diarrhœa will generally be found to be ulceration of limited extent low down the tube, and capable of being quickly and effectually removed by a strong solution of the nitrate of silver." (pp. 87-8.)

Dr. Stokes's remarks on jaundice may be read even by the most accomplished practitioner with pleasure and instruction, though we cannot say we agree with him in regarding inflammation of the stomach and duodenum as the most frequent cause of that affection—an opinion derived from Broussais. It is not to be denied, however, that jaundice, in a greater or less degree, is one of the most prominent symptoms of duodenitis, and there is every reason to believe that inflammation of the stomach and duodenum is the most characteristic feature of the malignant yellow fever of the tropics. The epidemic which occurred in Dublin in 1826-7 bore the closest resemblance to yellow fever, indeed there can be little doubt of their virtual identity, and the same appearances of abdominal disease were observed on dissection. The cases which occurred in the Meath Hospital were described in a monograph by Drs. Graves and Stokes, and we need not here dwell on those portions of their lectures in which they are alluded to.

Some of the more obscure points in the pathology and treatment of hepatic diseases are ably commented on by Dr. Stokes, and illustrated by some very remarkable cases, among which is one of jaundice, arising from aneurism of the hepatic artery—another in which a distended gall-bladder was mistaken for an hepatic abscess pointing externally, and punctured accordingly. There are also some important surgical remarks on the mode of opening hepatic abscesses, in which it is shown that the presence of a distinct fluctuating tumour, even with discoloration of the integuments, affords no certain indication that adhesion has taken place between the layers of the peritoneum, so as to render a direct opening into the abscess safe. Dr. Graves has invented an operation by which all danger of effusion is obviated in such cases. He makes an incision through the integuments over the most prominent part of the tumour, carries it nearly down to the peritoneum, but not quite, and keeps the wound open by plugging it with lint. The irritation of the wound causes the formation of adhesions internally, and after some time the abscess bursts with perfect safety to the patient. Such a proceeding has the advantage of being applicable to abscesses which have not yet distinctly pointed, and has a great effect in determining that occurrence by removing external resistance. We regard this as a most scientific and masterly operation, and would particularly recommend it to the attention of those (we hope they are now few) who regard a knowledge of surgery as unnecessary or derogatory to a physician. The cases in which the operation has been practised, as well as most of the other remarkable ones cited by Dr. Stokes in relation to diseases of the liver, have been published in the reports of the Meath Hospital.

Dr. Stokes notices a form of disease which is liable to be confounded with acute inflammation and abscess of the liver.

"There is one disease more which may be, and I believe has been, confounded with acute hepatitis and abscess of the liver. This affection, which has not been sufficiently noticed by authors, is inflammation and abscess of the abdominal parietes over the hepatic region; and this is a very singular disease. It is sometimes trifling, but I have seen a patient die of it. With the original nature of this disease I confess that I am not at all well acquainted; nor can I say whether the inflammation first attacks merely external parts, or whether it is a primary affection of the liver, and that the external parts take on diseased action from sympathetic irritation. In such cases we frequently observe many of the symptoms of inflammation of the liver, as pain, tenderness, biliary derangement, foul tongue, and morbid stools, with a tumefied state of the integuments. After these symptoms have continued for some time, the tumour increases in size, becomes softer, and matter forms. You give exit to the pus by opening the abscess with a lancet, and the patient gets well. This occurrence I have frequently witnessed. From a consideration of all the circumstances, it strikes me that in this disease the first morbid action in all probability commences in the liver itself, and that the external inflammation is an example of the strong sympathy which subsists between disease of deep-seated parts and integuments which cover them. Of this fact you have several illustrative instances. In pleuritis we frequently find the integuments of the chest remarkably tender on pressure; and in cases of inflammation of the brain the integuments of the scalp have their sensibility much increased. The same thing occurs in hepatitis; and in this disease one of the first distinct symptoms is this tenderness of the superincumbent skin. Now, you can conceive that, if this morbid sensibility of the investing parts should increase, in place of having some pain and tenderness, accompanied by swelling, we may have suppurative inflammation set up in these parts; and that, under such circumstances, the inflammation may leave the internal organ where it first existed, and be thrown upon the external parts in its vicinity. It strikes me that this is not unfrequently the case in this curious affection. In the case of this disease which I have seen prove fatal, the following circumstances were observed:—evident symptoms of inflammatory fever; pain and tenderness in the region of the liver, followed by the appearance of a tumour; which became fluctuating, was opened, and a quantity of matter discharged with considerable relief to the patient. She left the hospital, but returned again in about a fortnight or three weeks, with an enormous tumour in the same place, which was again opened, and a vast quantity of purulent matter evacuated. Though the matter continued to flow out freely, she did not recover strength; and on enquiry it was found that before her second admission she had spit up some blood. One day, while dressing the abscess, the gentleman who attended her observed that when she coughed air passed out through the wound, proving the existence of a fistulous communication with the lung. On examination after death we found an abscess, the base of which rested upon the peritoneal surface of the liver, without engaging its substance. From this the matter had made for itself a double passage, one externally, the other through the diaphragm and pleura into the substance of the lung. This was the only case in which I have seen this disease prove fatal; and in it death appears to have been caused by the extent of the disease, and by the abscess opening into the pleura and lung. (*Lecture* xvi., pp. 138-9.)

Such affections bear an evident analogy to that in which the abdominal integuments over the cæcum become inflamed in consequence of a corresponding state of that intestine. A description of the last-named affection has been very properly introduced by Drs. Bright and Addison. We have noticed it fully in a preceding article.

But we must here bring our remarks on abdominal diseases to a close. We have been tempted to carry them to a considerable length by the excellent materials which Dr. Stokes's lectures afford. Independently of the original thought and observation which these lectures display, they would be of the highest value if regarded only as an admirable critical exposition of the most important part of Broussais's doctrine.

*Diseases of the Circulating and Respiratory Organs.* The pathology of the thorax is perhaps more advanced than that of any other region: yet our knowledge in this department is quite of recent growth; for less than a century ago the real nature of phthisis was unknown, the diseases of the heart scarcely recognized, pulmonary hemorrhage referred exclusively to the rupture of vessels, hydrothorax treated of as an idiopathic disease, and bronchitis known only as a peculiar affection of old age!

The rapid improvement in this department of pathology appears to be chiefly owing to the favorable and united relation which physiology and morbid anatomy have preserved towards it for a series of years. While the footsteps of disease in the thoracic viscera have been followed with unwearied assiduity both in France and England, the physiology of the circulating and respiratory systems has been cultivated with zeal and success by enquirers of all nations: thus the two great sources of pathological knowledge have blended in a powerful stream. Yet even here we have but too much reason to acknowledge the imperfection of medicine and the absence of any uniform relation between the science and the art; for despite our scientific knowledge of thoracic diseases, they are much less under the control of therapeutical agents than many others of whose nature we are comparatively ignorant.

In the few remarks we are about to make on the pathology of the *heart*, we shall not dwell on its particular organic lesions. A minute description of these is not to be expected in elementary treatises, and our authors in general are not diffuse upon them. Dr. Elliotson's lectures contain a good deal of information relating to them, but it is delivered in too off-hand a manner for so intricate and controverted a subject, and difficulties are sometimes too unceremoniously put aside.

Among the inflammatory affections of the heart, *pericarditis* has received its due share of attention from our authors; and if hardly anything is said of *endocarditis*, it must be admitted that there is not much to say, since our knowledge of the subject is very incommensurate with its importance, and the diagnosis of endocarditis from inflammation of the pericardium cannot be formed with any degree of certainty. Our limits will not allow us to enter on these topics, but we cannot pass by the subject of the heart without briefly noticing a very universal yet unaccountable oversight, which at length seems to have been perceived all of a sudden; for several recent writers have alluded to it in one shape or another almost simultaneously. It is this: while the pulse has long been regarded as an index to the state of every system and organ of the body, it has been in a great measure neglected as an index to the state of that very organ of whose action it is the immediate result—namely, the *heart.* Hence great practical errors have arisen in regarding certain states of the pulse as denoting certain conditions of the general system, when they are in reality principally or solely referrible to the condition of the central organ of the circulation. This truth has lately been placed in a prominent point of view by Dr. Hope, who appears to have entered on the investigation with his usual assiduity, and from whose enquiries we anticipate some valuable results.

In a late review of Dr. Holland's "Medical Notes and Reflections," we took occasion to point out the great importance, in cases of apoplexy, of discriminating the hard pulse, which denotes an entonic condition of the system from the jarring pulse of cardiac disease. We would here extend the principle of this remark to another malady, to which its application may at first appear less obvious—we mean *acute rheumatism.* If we consult practical writers as to the state of the pulse in this disease we shall find an extraordinary discrepancy. To go no further than the authors before us—Dr. Elliotson tells us that the pulse is soft and full (p. 738); Dr. Gregory that it is full, soft, and as it were *round* (p. 673); Dr. Reid says it is large, full, and hard (p. 413-15); and Drs. Bright and Addison that it is full, hard, and jerking (p. 562-83). All of course agree that it is frequent. The truth is, the pulse is various: nevertheless we think that there is a pulse generally characteristic of rheumatic fever, at least in its early stage; but we cannot regard any of the above descriptions as conveying an accurate idea of it. We should say that it was full, *tense*, and occasionally somewhat thrilling. It does, however, present a like variety with the pulse of nervous palpitation, and, we imagine, is influenced by the same cause—a morbidly irritable state of the heart. The opinion of Bouillaud that pericarditis or endocarditis exist in the majority of cases of acute articular rheumatism we hold to be altogether extravagant. If this were true, rheumatism would be a very fatal disease, which every one knows it is not, and patients would not recover under the most opposite modes of treatment as we daily see them doing. Nevertheless, the supervention of inflammatory affections of the heart is frequent enough to show that this organ is peculiarly liable to become implicated in the rheumatic state. But to the best of our observation the excitement of the circulation in rheumatic fever is usually more than commensurate with the other signs of inflammatory action throughout the system, and liable to variation from circumstances that would have little effect if it resulted solely from an inflammatory cause; and we have occasionally found that the full and tense state of the pulse has been increased by large bloodletting: from all which we infer that independently of the inflammatory condition of the membranes which often supervenes, an irritable state of the muscular fibre of the heart is a prominent feature of rheumatic fever.

Much misconception with regard to metastasis has arisen from the neglect of auscultation. A patient is affected with acute articular rheumatism; the disease in the external parts subsides, but instead of the hoped-for recovery it is found that there is disease of the heart: in these circumstances it is said that metastasis has taken place, while, in reality, the pericardium or endocardium has been affected from an early stage of the disorder, if not from its commencement, but this important fact has been overlooked; the rheumatism, having run its course, has disappeared altogether in the external parts, because it has there no tendency to produce organic change, but it has left a fatal record of its presence in the very centre of life. We have no doubt that all this is familiar to many practitioners, but it has not hitherto been taught in the schools, and we have therefore great pleasure in directing the attention of the student to the following extract from the work of Drs. Bright and Addison, which we believe to contain a very correct view of the subject:

"The organs and parts which have been observed to be affected with inflammation in connexion with rheumatitis, are the heart, the brain, the lungs, the stomach, the bowels, and the sclerotic coat of the eye. Indeed, a morbid condition of the heart is so very familiar a complication in rheumatitis, that it appears to be more correct to regard it as constituting a part of the rheumatic disease than as an accidental and only occasional occurrence from metastasis. The morbid condition of the heart, nevertheless, is by no means uniform in its kind. In almost every case of acute rheumatism, we find the organ remarkably irritable, as indicated by the violence of its action, and by the facility with which it is thrown into a state of considerable excitement; a degree of excitement sufficient in some instances, especially after copious depletion or in chlorotic subjects, to produce a distinct *bruit de soufflet*, calculated to mislead to a belief in the existence of some more serious lesion. This morbid irritability is, however, of little moment, and usually subsides as the disease is subdued by appropriate remedies. In other cases, but much more rarely, the heart is liable to be seized with a severe crampy or neuralgic pain, which proves exceedingly distressing to the patient, and is compared to a grasping or squeezing of the organ: this is for the most part of short duration, and generally yields speedily to moderate depletion. A much more common, and unfortunately a much more serious affection, is that which is regarded as the ordinary metastasis to the heart. This consists in acute inflammation of the external or internal membrane of the heart, or of both of these structures at the same time, constituting accordingly pericarditis or endocarditis or both. These complications are by far more common than is generally supposed, and are very frequently altogether overlooked, in consequence of the obscurity of the symptoms to which they give rise in many cases. The student ought at all times, therefore, to be attentively on the watch; and by taking alarm at the slightest indication of disturbance of the heart, and by a judicious application of auscultation, endeavour to steer clear of that which must at all times prove a most serious, and not unfrequently a fatal oversight; always bearing in mind also, that in proportion to the youth of the patient, is the complication more likely to take place." (pp. 564-5.)

Not only is it true that certain pathological conditions of the heart itself may so affect the pulse as to lead to erroneous impressions as to the general state of the vital powers, but it is true also that the condition of the heart, as remotely influenced by disease of the brain, may lead to similar errors. This is well illustrated by Dr. Graves, who gives a striking case of acute disease of the brain consisting, as appeared on dissection, in sanguineous effusion, ramolissement, and extensive suppuration, attended with great arterial excitement and a hard pulse, in which, nevertheless, the abstraction of fourteen ounces of blood was followed by sudden sinking of the pulse and death in about two hours. (p. 53.)

We must notice yet another point relating to the heart which is neglected by the majority even of good practitioners, to wit, the importance of attending to the *natural condition of the organ*, which varies considerably in different individuals. Bouillaud, Bizot, Clendinning, and others have been at much pains to ascertain the mean size and weight of hearts, the mean thickness of their parietes, &c., and the observations of Bizot are particularly valuable as tending to establish a difference of dimension at different ages. But in this, as in many other instances in which arithmetic is applied to medicine, we arrive after all at a mere abstraction: thus the mean thickness of fifty hearts may be represented by a number which does not indicate the actual thickness of any one of them; and in our anxiety to establish this mean, we lose sight of the *diversity of the individual hearts*, which is a matter of much more practical consequence. This remark will not appear frivolous if we reflect how small a difference in the thickness of the ventricular parietes will make an appreciable difference in the sound and impulse, and how small in truth is the actual difference of thickness between a healthy ventricle and one in a state of incipient hypertrophy. Now, in examining a patient's heart, we may find a feeble impulse and a somewhat greater extent of sound than usual, and may hence be led to suppose that there is a degree of passive dilatation resulting from disease; but in many individuals the parietes of the heart are naturally attenuated and its muscular power comparatively small—conditions which will often be found in conjunction with a general laxity of fibre and a weak and flabby state of the muscles throughout the body. On the other hand, the walls of the heart may be naturally strong and thick, the impulse powerful, and the sound limited. In such cases as these we are not to infer from slight deviations of the impulse or sound from the ordinary standard, that there is disease of the heart or even a tendency to it. Yet we affirm with submission, but at the same time with confidence, that we have known celebrated auscultators led into gross mistakes, and much needless anxiety occasioned to patients and their friends, by want of attention to these congenital peculiarities in the structure of the heart. A patient has palpitation from some sympathetic and transient cause; the auscultator detects a somewhat greater or smaller strength of impulse or extent of sound than is normal, and hence infers incipient organic disease; whereas if he had been previously in the habit of examining the action of the heart in this patient, he would know that matters had never been otherwise. Shall we be forgiven for insinuating that under such circumstances the fancy sometimes gains an ascendant over the hearing; divers *bruits* which have no existence are recorded, and the patient is frightened out of his wits because his heart is—as nature made it. Every practitioner, therefore, who is in the habit of attending a patient, should make himself minutely acquainted with the natural peculiarities of the heart as well as those of the pulse.

For a correct and comprehensive general view of the diseases of the *respiratory organs* we may refer the student particularly to the work of Drs. Bright and Addison, who appropriately introduce the subject by some observations on the different forms of catarrh. It is curious with respect to the treatment of the ordinary acute form of this affection, that while the copious use of diluents is commonly recommended, Dr. B. C. Williams has found a remedy in a total abstinence from liquids for thirty-six or forty-eight hours. Dr. Reid states that he has adopted the latter plan with success, and we have heard the same statement from several intelligent practitioners.

In treating of *acute bronchitis*, it is usual to represent the extent of membrane affected as the principal measure of the danger of the case. We think, however, that this is not quite a correct view of the subject, and that much more depends on the situation and physiological relations than on the extent of the inflamed surface. The mucous membrane may be inflamed throughout the larger bronchial tubes, and yet the patient shall not be seriously ill; but the moment the disease extends to the smaller ramifications it becomes dangerous: in the former instance we have merely inflammation of a certain extent of membrane which is not implicated in the performance of any function immediately essential to life; but in the latter instance respiration is impeded by the tumefaction of the membrane in the minute tubes, and the effusion from its surface. In a great majority of fatal cases of bronchitis, the real cause of death is asphyxia; the patient is said to *sink*, but he is in fact suffocated. Want of attention to this important difference in the anatomical relations of the respiratory membrane in different parts of its course not only blinds us to the chief source of danger, but leads to discrepant and erroneous views of practice. It is currently said that bronchitis will not bear full bleeding; Dr. Elliotson, however, tells us, with reference to common acute bronchitis, that we "have only to bleed the patient well and follow it up by local bleeding." Dr. Reid also recommends the free use of the lancet. Dr. Gregory remarks that "the urgency of the symptoms appears to demand the copious abstraction of blood, but the constitution seldom bears it well." Drs. Bright and Addison, considering that the inflammation is in a mucous membrane, and that inflammation in this tissue is little under the control of depleting measures, generally running a course of indefinite duration in spite of remedies, though it be subdued in degree—that the morbid secretion obstructs the bronchi, and seriously interferes with the functions of the lungs—and that the patient has, therefore, to pass through a longer or shorter period of exertion, distress, and restlessness—arrive at the conclusion "that all remedies calculated greatly to impair the patient's strength, should be employed with the greatest reserve, and that even in the most acute attacks, the period of active depletion is likely to be of very short duration." According to our own observation when the inflammation is situated in the larger tubes, and the patient's constitution is firm, bronchitis bears bleeding perfectly well, and this remedy may be applied under the same regulations as in any other disease; but when effusion is going on rapidly in the minute bronchial tubes, the case is widely different; every organ and tissue of the body is suffering from deficient aëration of the blood, and all the powers of life require to be supported and stimulated: in addition to this we may remark that although bloodletting be a powerful means of arresting effusion from the surface of serous membranes when acutely inflamed, there are many cases in which it increases the disposition to morbid secretion—as, for example, where suppuration has commenced in the parenchyma of the lungs or liver; and in this very case of increased mucous secretion into the minute bronchial tubes.

It is not our intention to dilate on those numerous affections of the air-tubes in which spasmodic action and vascular derangement are liable to be so blended or alternated, that the opinions of pathologists have long been, and probably will long continue to be, divided as to their essential nature, and as to the legitimacy of specific distinctions among their various forms. We may, however, remark, that if we consider on the one hand that the pneumogastric nerves appear to have a powerful influence on the action of the vessels throughout the lining membrane of the air-passages, and on the other hand that the whole respiratory system of nerves are engaged in various and constant reflex actions, so that irritation of the membranous surface readily produces spasm or other morbid condition of the muscular parts of the apparatus, it is easy to understand that there are numerous cases of disease in which it is nearly impossible in a pathological, and not very important in a practical, point of view to determine whether nervous or vascular derangement have generally the precedence—seeing that in a case of any duration both must almost inevitably be present, and the treatment must be regulated according to the actual predominance of one or the other. We may cite as examples *croup and asthma.* With respect to spasmodic croup, or *laryngismus stridulus,* we think that the opinion of the late Dr. Ley, as to its peculiar pathology, has made sufficient impression to entitle it at least to notice in elementary works on the practice of physic. None of our authors, however, make any allusion to it except Dr. Reid, who says that the malady "is more generally believed to arise from a state of irritation of the eighth pair of nerves, depending on a diseased state of the contiguous glands, the cervical and bronchial." But here too much influence appears to be assigned to Dr. Ley's doctrine, which we do not think is generally received, and which, in its full extent, we are certainly not disposed to admit, because, although scrofulous children may in general be most subject to the disease in question, we frequently meet with it in children who present no indications of this diathesis, in whom there is no disease in the cervical glands, and no reason to suspect it in the bronchial. But we do not consider that part of Dr. Ley's hypothesis which relates to the glands as the most important; it is the notion of the paralyzed state of the muscles of the glottis as opposed to that of spasm which, in our opinion, constitutes the point most worthy of consideration.

The diagnosis of *pneumonia* is ably commented on by Drs. Bright and Addison, who very truly observe that it is by no means so easy as some persons have imagined. They dwell especially on pungent heat of the surface as the most invariable and conclusive diagnostic, and think that where inflammation *is confined to the chest,* however various may be the textures involved, the presence of this symptom may be taken as a certain indication of pneumonia in nineteen cases out of twenty. We believe this opinion to be just with reference to common pneumonia, and it is doubtful whether the disease called *typhoid pneumonia*—in which the skin is cool—forms any real exception, since there is some reason to believe that the pneumonia in this case is merely a complication of a malignant fever, of which enteric inflammation also forms a prominent feature. Their account of the physical signs of pneumonia is excellent: the following description of the mucous rattle, as heard through the medium of a hepatized portion of lung, though the similies be anything but poetical, will strike the reader accustomed to auscultation as particularly true to nature.

"The mucous rattle attendant on pneumonic consolidation becomes, in some instances from the latter circumstance, remarkably striking and characteristic, each inspiration conveying to the ear of the auscultator a sound resembling that produced by raising a flat fish from a moist and unctuous slab; or perhaps a sound so coarse, as rather to resemble the whizzing and gurgling occasioned by a cow suddenly raising its flat foot out of a mass of mud." (pp. 239-40.)

Dr. Graves makes a remark on percussion as diagnostic of solidification of the lung, which we believe is new—namely, that although the positive evidence of condensation of the lung afforded by percussion may be depended on, the absence of such evidence is no absolute proof that the lung is not solidified. He states that he has met with several instances in which percussion elicited a very clear sound from the parietes of the chest, where there was considerable solidification of the lung within. The following case he thinks affords a solution of the phenomenon:

"An old man, named Foy, died lately at Sir P. Dun's Hospital, of hepatization of the inferior lobe of the right lung, with numerous tubercular depositions in the upper lobes of both lungs. During his illness, I pointed out the existence of extensive hepatization of the lower lobe of the right lung, in which perfect and decided dulness marked out accurately the space occupied internally by the solidified pulmonary tissue. But anteriorly and above, the parietes of the chest returned a clear sound on percussion, nor could a vestige of dulness be anywhere detected. Yet the whole of the upper lobes of this patient's lungs were occupied to such an extent by crude tubercles, that no portion of the upper lobes could be selected, equal to half the size of a fist, which would not sink in water. This was owing to tubercular matter, which occupied the pulmonary tissue in detached infiltrated masses, or in single crude tubercles. How, then, did it happen that such extensive solidification of the upper lobes existed without any corresponding dulness on percussion? A careful examination of the pathological condition of these lobes satisfactorily explained the anomaly. On accurate inspection, we found that although the solidified masses of the pulmonary tissue were extremely numerous, and predominated over the parts which still retained their natural vesicular texture, so that an extensive portion of the upper lobes seemed to be quite solid, yet the solidified portions were insulated and divided from each other, throughout the interior of the lobe, by intervening laminæ of healthy pulmonary tissue, and on their surface were, for the most part, covered by a stratum of healthy vesicular lung, from a quarter to half an inch in thickness. Indeed, although the solidified masses (to use a geological expression) sometimes cropped up, and came to the surface, yet this was comparatively a rare occurrence; and by far the greater portion of that surface was composed of a thin stratum of pervious vesicular tissue. To this was owing the clear sound elicited by percussion. You will recollect, therefore, that in certain (I will admit rare) cases of tubercular deposition in the lungs, the tubercular development may have proceeded to the extent of rendering the greater portion of the upper lobes impervious to the air, and may have solidified those lobes considerably, and yet the solidified portions may be so divided from each other by laminæ of healthy lung, and may be so covered by a stratum of vesicular tissue, that the general result of percussion is to elicit a clear sound over the whole of the parietes of the chest corresponding to the affected lobes."(pp. 355-6.)

On the treatment of pneumonia we quote the following from Dr. Elliotson, because it actually contains all he says about the matter, and presents a characteristic specimen of the summary style in which he sometimes disposes of a subject:

"It is only the treatment of any inflammation. Patients have sometimes borne the loss of an immense quantity of blood; perhaps more in this disease than in most others. It is in this affection that those enormous bleedings are reported to have taken place which I mentioned when speaking of inflammation in general—a few gallons in the course of a few days. Mercury is of the same use in this affection as in bronchitis, and in bronchitis as in other inflammatory diseases." (*Ed. Cooke and Thompson*, p. 503.)

We have been accustomed to consider pneumonia as perhaps the one of all purely inflammatory diseases which most severely exercises the judgment of the practitioner as to the precise point to which bloodletting ought to be carried; for there is assuredly, in many cases, a period at which the inflammation, though diminished by active depletion, continues in sufficient force to threaten a fatal result, but where, nevertheless, the further use of bloodletting would only hasten disorganization. Under these circumstances, we conceive it is a matter of some importance to determine whether there be any means which may take the place of bloodletting, when this ceases to be applicable. It is somewhat remarkable that neither Dr. Elliotson nor Dr. Gregory so much as mention the contra-stimulant treatment. Dr. Reid observes that this practice has been so much less successful in England than in France as to lead to the supposition that there must be something different in the character of pneumonic disease in the natives of the two countries. We apprehend, however, that the comparison of this with the ordinary antiphlogistic treatment has been made from data somewhat different in England and on the continent. We think it may be said, without prejudice, that the lancet is used more vigorously, and at the same time more cautiously, with a stricter regard to scientific principles, and consequently with greater success, in England than in most other countries. We can, therefore, easily conceive that a comparative estimate of the effects of bloodletting and emetic tartar might lead to conclusions more favorable to the latter in France or Italy than in this country. Dr. Reid states that he should, "from considerable experience of both modes of treatment, be undoubtedly inclined to avoid it [the contra-stimulant] altogether, except in those cases where the use of the lancet could not be practised." (p. 328.) This is rather ambiguously expressed. If it be meant that tartar-emetic is useful in cases to which the lancet would from the first be inapplicable, we do not think the opinion just, for we believe that this medicine does good exactly in the same cases as the lancet; that is to say, where the state of the system is entonic and the crasis of the blood firm: if it be meant that the tartar-emetic may be serviceable where the lancet has been used as far as it could be with safety, we admit this to be true.

We must not omit to notice a new practice recommended by Dr. Graves, in the *scrofulous* form of bronchitis and pneumonia. From the success of Dr. O'Beirne's treatment of scrofulous inflammation of the joints, Dr. Graves was led analogically to try if similar inflammation of the lung might not be arrested by the bold use of mercury. The result of the experiment has been satisfactory; and he believes that he has often succeeded, by this practice, in checking incipient phthisis. We must confess we should have supposed this treatment of all others the most likely to precipitate the patient into phthisis. We admit, however, the insufficiency of *à priori* reasoning in the practice of medicine.

*Diseases of the Nervous System.* On these much valuable information is diffused through the volumes before us. The best connected view of them, we think, is that comprised in the second part of Dr. Gregory's work; and to this we must content ourselves with merely referring, since to enter with any effect into the details of such subjects would require a number of this journal instead of an article. Two very important general points have recently occupied a large share of the attention of philosophical physicians: the one is the existence of the same morbid state of the cerebral functions in connexion with the most opposite conditions of the vascular system and of the vital force; the other is the reflection of nervous actions through the medium of certain portions of this system. The illustration of the former of these subjects, and of the latter also in its pathological relations, is almost exclusively due to English writers.* Their further prosecution will probably lead to material changes both in the theory and treatment of nervous disease.

We regret much that the portion of Dr. Stokes's lectures which we have received breaks off soon after he commences the subject of nervous pathology. His introductory observations are admirable. On the nature of the pure *neuroses*, or those diseases of the nervous system in which the most careful examination after death can detect no structural change in any of its parts, two opposite opinions have been held: the one ascribes them to some peculiar modification of nervous influence, or, in other words, some alteration in the manifestation of the vital power, independent of any molecular change; the other refers them to molecular changes which are so minute as to elude our research. The following remarks of Dr. Stokes contain an analogy so new and ingenious, that we cannot do better than lay them before the reader in his own words:

"You must have been already convinced that it is difficult to form any clear or definite notion of the nature of neuroses; indeed, the only thing we can say of them is, what they are not. When we reflect on nervous phenomena, and consider how occult, how mysterious, the properties of those organs which give rise to them are, we are struck with astonishment at the discrepancy between cause and effect. No medical man has ever witnessed a case of confirmed tetanus or hydrophobia, without being oppressed with a conviction of the imperfect and limited state of our knowledge of nervous disease. It may be very possible, that in these neuroses the change, though so slight as to escape our means of detection, does absolutely occur; and yet such is the nature of nervous phenomena, that we must admit that great and extraordinary effects are produced by very slight causes. Do we see anything like this in nature?—any remark-

* Principally Whytt and Marshall Hall.

able alterations in properties depending upon apparently slight causes? We do—we see extraordinary changes taking place in the characters of various inorganic substances, (to which I need not particularly allude,) and there is no reason why the same thing should not occur in organic structures. On considering the doctrine of Isomerism, I should be inclined to think that it throws some light on this obscure subject. In chemistry, it is a well-known though singular law, that the properties of two bodies may be essentially different at the same time that their respective component elements are, as far as our knowledge goes, identically the same; and the change, whatever it may be, appears to result, not from the abstraction or removal of any of the component atoms, but from their peculiar juxtaposition. Now, it being admitted in chemistry that many bodies having the same constitution possess totally different properties, and this difference being explained by the different position of their elements, it does not seem strange if the same thing should take place in the phenomena of organized beings; and, if this be the case, we have a key towards elucidating the nature of these neuroses, and can conceive how an analogous change—a difference in the arrangement of the molecules of the component parts of the nerves, or their centres—may produce new modifications of their properties, without making any distinct change in their nature, or adding or abstracting a single organic molecule. I am much inclined to adopt the opinion of those who think that, in the neuroses, a peculiar organic change actually takes place, though we cannot demonstrate its existence; because, to reason on the phenomena of animal life, independently of organization, is to plunge blindly into hypothesis, and retrace the errors of an antiquated and exploded school." (pp. 198-9.)

But we must not indulge further in theory: we proceed, therefore, to the last subject which it is our intention to notice, namely, *Diseases of the Spinal Cord.* These we advert to chiefly to express our regret at the extraordinary neglect they have met with in this country. While treatises on spinal distortion are appearing every day, and the somewhat intangible subject of "spinal irritation" has received of late more attention than is perhaps due to it, the inflammatory and organic affections of the spinal cord and its membranes are almost entirely overlooked. Except the valuable contributions of Dr. Abercrombie, and a good article by Dr. Todd, in the Cyclopædia of Practical Medicine, there is, we believe, nothing like a treatise on the pathology of the spinal cord in the English language, and the greater part of our elementary writers on the practice of physic seem to have excluded it as by common consent. The diseases of this organ are not indeed very frequent, but they are quite as much so as some others on which it is usual to comment. Among the writers before us, Dr. Reid has devoted a page and a half to "arachnitis spinalis," but it is not very clear which of his remarks apply to the acute and which to the chronic form of the disease; nor do we think they can convey to the mind of the student any very distinct notion of either. Dr. Graves gives the details of two remarkable cases of ramolissement of the cord connected with the retrocession of gout from the extremities. He intimates his belief that gouty inflammation may seize on the nerves and neurilemma, and be propagated along their course to the spinal cord and its sheath. This he conceives to have occurred in the cases alluded to, though we are not sure that he has succeeded in making out the point distinctly. In favour of the existence of a gouty affection of the spinal cord, he adduces some cases recorded by Dr. Copland and Dr. Prichard; he has, however, fallen into a mistake with respect to Dr.

Prichard's cases, which were not rheumatism but *chorea*, or an affection resembling it.*

It will be thought incumbent on us, in taking leave of our authors, to pass some opinion on the general merit of their respective works; and it is very gratifying to us to be able to speak with high commendation of them all. It is but justice to Dr. Elliotson to say, that we know not how far the rival editions of his lectures contain a faithful transcript of the originals. Dr. Rogers's book may be considered as a work of his own, founded on Dr. Elliotson's lectures, rather than as an edition of the latter. There is no division into separate discourses, and the editor states in his preface that he has altered the phraseology throughout: he has prefixed some introductory remarks, likewise altered, from the author's Lumleyan lectures; and he has added an appendix, derived from heterogeneous sources, which we think might as well have been omitted. We consider this parasitical species of editing as extremely objectionable, except in the case of standard works which have become more or less obsolete, and require remodelling to adapt them to the actual state of knowledge. No one can suppose that Dr. Elliotson's lectures stand in this predicament. In the edition by Drs. Cooke and Thompson, the lectures are preserved in their distinct form; and we presume that they do not differ materially from the originals, since we trace in them the terse and facetious mode of expression which characterized those lately delivered by the author, and which, together with their fulness of information, rendered them deservedly popular. The work of Drs. Bright and Addison, as far as it has yet proceeded, fulfils admirably the intention with which it was undertaken, and we know of none which we can more strongly recommend as a text-book for students. The subjects flow easily into each other, and the information is accurate and copious, without too much minuteness of detail. Dr. Reid's book, though not tastefully written, evinces much pathological and practical knowledge, and contains many sagacious remarks. We have seldom met with a work which condensed so much valuable matter in the same space. Dr. Gregory's "Elements," though rather too concise on some points of pathology, are very uniformly good: the practice recommended is for the most part highly judicious, and the style of writing is correct and perspicuous. The lectures of Drs. Graves and Stokes, though now presented for the first time to the public in a connected form, are already too well known to need any commendation from us. Our frequent reference to them in the course of this article is a sufficient proof of the estimation in which we hold them.

On comparing the works which we have been examining with each other, and with the majority of those which have lately issued from the press, it is encouraging to perceive a great increase of unanimity in the view of authors, especially with respect to the more important points of practice. This may be regarded as a clear indication of an improved method of observing, and as an earnest that something like certainty may yet be hoped for in medicine, despite those who sneer at its fallacious evidence, its changeful theories, and its meager resources.

* See Medical Repository, vol. xxi.

